# Metal-free initiators pave the way for chemically recyclable polymers with industrially relevant properties

**DOI:** 10.1038/s42004-023-00931-4

**Published:** 2023-06-19

**Authors:** Ainara Sangroniz, Haritz Sardon

**Affiliations:** POLYMAT, University of the Basque Country UPV/EHU, Joxe Mari Korta Center, Avda. Tolosa 72, 20018 Donostia-San Sebastian, Spain

## Abstract

To solve the environmental disaster that is generated by legacy plastics accumulation, researchers are looking to design plastics with enhanced end-of-life options, but many circular plastics do not meet industrial requirements. Here, we highlight a metal-free approach to produce chemically recyclable poly(1,3-dioxolane) with ultra-high molecular weight and comparable properties to one of the most produced plastics, polyethylene.

One alternative to transition from our current linear plastic production to a more sustainable circular plastic production is to design plastics capable of chemical recycling to monomers (CRM)^1–5^. CRM mitigates the need for continuous feedstock sourcing and could potentially minimize the accumulation of plastic waste. While initial reports on CRM plastics did not specifically focus on the potential scalability of these materials, in recent years, special attention has been paid to the design of easily scalable systems, accessing useful material properties, and invoking simple processes for depolymerization to monomers. Polyacetals are promising candidates for CRM and, recently, a reversible-deactivation cationic ring-opening polymerization (CROP) of cyclic acetals using a commercial halomethyl ether initiator and an indium(III) bromide catalyst was introduced^6,7^. Using this method, poly(1,3-dioxolane) (PDXL) of around 200 kDa was produced, obtaining materials with tensile strengths comparable to some commodity polyolefins that could be chemically recycled in near-quantitative yield in the presence of strong acids. So far, sustainable catalysts/initiators that could transform CRM plastics into ultra-high molecular weight plastics with unique toughness thanks to their superior entanglement have been lacking.

Now, H. Grace Hester, Brooks A. Abel, and Geoffrey W. Coates from Cornell University, USA, employ commercially available Meerwein salts as organic initiators together with 2,6-di-tert-butylpyridine (DTBP) as a proton trap to synthesize ultra-high molecular weight PDXL of up to 2000 kDa from recycled DXL (10.1021/jacs.3c01901)^8^. The oxonium (Meerwein) salt initiators with non-nucleophilic counter-anions generate small concentrations of propagating cations. “We realized that transacetalization, a side-reaction during CROP of cyclic acetals, could be used as a mechanism for sharing propagating cationic species among different polymer chains,” says Abel. “This means that even if a propagating polymer chain terminates, this transacetalization ‘side-reaction’ revives the dead chain by transferring the propagating cation from a living chain to the dead chain. This key insight was crucial to developing the present cyclic acetal CROP system”. Because of the living nature of the polymerization, extremely low initiator concentrations i.e., monomer to initiator ratios of up to 40,000: 1 suffice for initiating the CROP of cyclic acetals. “We showed that the use of DTBP as a proton trap during the CROP of cyclic acetals prevents undesired initiation by acidic impurities and is crucial to achieving the molecular-weight control needed to reach ultra-high molecular weights,” says Abel. The team found that the increase in molecular weight leads to a tremendous improvement of the physical performance with maximum values for tensile stress at break of 105 MPa. The material is ductile and tough and shows a higher ultimate stress tolerance than ultra-high molecular weight polyethylene (UHMWPE) (molar mass ∼5000 kDa). “Our method of repolymerization of recycled DXL enables the synthesis of pristine PDXL with different molecular weights, mechanical properties, and architectures without affecting future recycling of the polymer,” explains Abel.

Industrially viable plastics capable of CRM could bring a paradigm shift to solve issues arising from the current unsustainable plastics production system. Proper catalyst/initiator selection may well facilitate the development of such unique sustainable polymers. Furthermore, chemical diversification in the field of CRM is crucial to cover the many applications where legacy plastics are present. Other physical aspects such as molecular weight are also critical to enhance the field of plastic materials. “We are now taking steps to synthesize polyacetals of varying polymer architectures to enable further tuning of polymer properties to fit a particular application,” says Abel.
